# Assessing the Viability of Social Media for Disseminating Evidence-Based Nutrition Practice Guideline Through Content Analysis of Twitter Messages and Health Professional Interviews: An Observational Study

**DOI:** 10.2196/jmir.5811

**Published:** 2016-11-15

**Authors:** Rosa K Hand, Deric Kenne, Taylor M Wolfram, Jenica K Abram, Michael Fleming

**Affiliations:** ^1^ Academy of Nutrition and Dietetics Chicago, IL United States; ^2^ College of Public Health Kent State University Kent, OH United States; ^3^ Antidote Education Company Dallas, TX United States

**Keywords:** social media, information dissemination, medical nutrition therapy, evidence-based medicine, heart failure

## Abstract

**Background:**

Given the high penetration of social media use, social media has been proposed as a method for the dissemination of information to health professionals and patients. This study explored the potential for social media dissemination of the Academy of Nutrition and Dietetics Evidence-Based Nutrition Practice Guideline (EBNPG) for Heart Failure (HF).

**Objectives:**

The objectives were to (1) describe the existing social media content on HF, including message content, source, and target audience, and (2) describe the attitude of physicians and registered dietitian nutritionists (RDNs) who care for outpatient HF patients toward the use of social media as a method to obtain information for themselves and to share this information with patients.

**Methods:**

The methods were divided into 2 parts. Part 1 involved conducting a content analysis of tweets related to HF, which were downloaded from Twitonomy and assigned codes for message content (19 codes), source (9 codes), and target audience (9 codes); code frequency was described. A comparison in the popularity of tweets (those marked as favorites or retweeted) based on applied codes was made using *t* tests. Part 2 involved conducting phone interviews with RDNs and physicians to describe health professionals’ attitude toward the use of social media to communicate general health information and information specifically related to the HF EBNPG. Interviews were transcribed and coded; exemplar quotes representing frequent themes are presented.

**Results:**

The sample included 294 original tweets with the hashtag “#heartfailure.” The most frequent message content codes were “HF awareness” (166/294, 56.5%) and “patient support” (97/294, 33.0%). The most frequent source codes were “professional, government, patient advocacy organization, or charity” (112/277, 40.4%) and “patient or family” (105/277, 37.9%). The most frequent target audience codes were “unable to identify” (111/277, 40.1%) and “other” (55/277, 19.9%). Significant differences were found in the popularity of tweets with (mean 1, SD 1.3 favorites) or without (mean 0.7, SD 1.3 favorites), the content code being “HF research” (*P*=.049). Tweets with the source code “professional, government, patient advocacy organizations, or charities” were significantly more likely to be marked as a favorite and retweeted than those without this source code (mean 1.2, SD 1.4 vs mean 0.8, SD 1.2, *P*=.03) and (mean 1.5, SD 1.8 vs mean 0.9, SD 2.0, *P*=.03). Interview participants believed that social media was a useful way to gather professional information. They did not believe that social media was useful for communicating with patients due to privacy concerns and the fact that the information had to be kept general rather than be tailored for a specific patient and the belief that their patients did not use social media or technology.

**Conclusions:**

Existing Twitter content related to HF comes from a combination of patients and evidence-based organizations; however, there is little nutrition content. That gap may present an opportunity for EBNPG dissemination. Health professionals use social media to gather information for themselves but are skeptical of its value when communicating with patients, particularly due to privacy concerns and misconceptions about the characteristics of social media users.

## Introduction

The Academy of Nutrition and Dietetics published an Evidence-Based Nutrition Practice Guideline (EBNPG) for Heart Failure (HF) in 2008 [1]. Evidence supports the use of nutrition to manage the symptoms of HF and improve quality of life [1]. With these outcomes in mind, the EBNPG for HF provides recommendations on the use of medical nutrition therapy, sodium and fluid restriction, energy and protein needs, and dietary supplements [1]. These recommendations, along with all Academy of Nutrition and Dietetics (Academy) EBNPGs, are available on a website for Academy members and subscribers. Dissemination efforts focus on raising awareness of new guidelines among Academy members through email blasts and inclusion in newsletters to relevant subgroups of the organization. When the EBNPG for HF was developed in 2008, social media in its current form was relatively new (tracking by the Pew Research Center began in 2005), and therefore social media was not used for initial EBNPG for HF dissemination efforts [2]. More recent Academy EBNPGs are promoted through the Academy’s professional social media channels. Whereas the primary audience for Academy EBNPGs is registered dietitian nutritionists (RDNs), increasing knowledge of the EBNPG among physicians and patients may help to increase the implementation by increasing RDN consultations for HF.

The use of the HF EBNPG, which has 11 specific recommendations on HF and medical nutrition therapy, protein needs, energy needs, fluid intake, sodium intake, alcohol, and dietary supplements, including folate, vitamin B12, thiamine, L-Arginine, Carnitine, Coenzyme Q10, and Hawthorne, has been limited relative to the use of other Academy ENBPGs. For example, 29 HF EBNPG toolkits (a supplemental product to assist in implementing an EBNPG) were purchased in 2015, which is 62% of the average sales for each of the other toolkits during the same period (unpublished data). Similarly, the digital EBNPG for HF received 2425 page views (1040 unique visitors) in 2015 when compared with an average of 4841 total annual page views for other individual Academy EBNPGs. The EBNPG for HF was also available through the National Guideline Clearing house for the first 5 years after publication and received 5049 page views through guideline.gov during this time. The causes or reasons for low utilization of the EBNPG for HF are unclear but may include lack of awareness or the small volume of HF patients referred to RDNs. The age of the specific EBNPG may also be a reason for low utilization; its content is undergoing revision currently and an update will be published soon. One proposed strategy for increasing referrals is raising awareness among physicians, RDNs, and patients about the availability of the EBNPG for HF and its content through the use of social media.

Social media is widely used in the United States, which shows its potential value as a dissemination tool. The Pew Research Center reports that 90% of all young adults and 35% of adults aged 65 years and above use social media in the United States [2]. Although there are disparities based on income and educational achievement, a large number of sociodemographic groups are connected to social media, and thus disparities are decreasing [2]. The Internet, including social media sites, are frequent sources of health information and support. One in 3 Americans has gone to the Web to attempt to diagnose a medical problem and a quarter have read about another individual’s health condition or sought support on the Web from individuals with a similar condition [3]. Therefore, providing health information through social media may be a viable strategy for dissemination of evidence-based health care information to patients and professionals. This may be particularly important for breaking through the “noise” of nonevidence-based information available on social media. In a study of Web postings by Italians, Mazzocut et al [4] showed that patients frequently search for and post items related to alternative therapies for cancer treatment, many of which involve nutrition, showing that nutrition therapies are a topic of conversation on social media.

However, less is known about health care providers’ use of social media to gather or distribute information. Other authors have reported that, in general, physicians’ willingness to use social media for professional development is based on ease of use and attitude toward social media (ie, for nonprofessional development activities) [5]. Previous research has identified 6 benefits and 12 limitations for the use of social media in health communication with benefits that include reducing stigma and collecting data on patient experiences and opinions and limitations, which include lack of reliability, quality concerns, and lack of privacy and confidentiality [6]. Most of the work in understanding health care providers’ use of social media is related to the privacy and ethical concerns that surround its use [7].

Social media is characterized by interactivity and user-generated content [8]. Therefore, the content is driven by those who choose to participate in social media and does not include content from those who are reluctant to engage, potentially impacting health care professional voices on social media. Popular social media channels include Facebook and Twitter, which is a micro-blogging platform with a limit of 140 characters per message (tweet). Twitter encourages content classification and interactivity through a variety of features, including hashtags (#) and addressing public tweets to specific user(s) (@). Hashtags are user-generated meta-data that allow a searchable grouping of related tweets. Use of the “@” symbol plus a username provides the ability to specifically target a message to a specific user(s). Finally, retweeting or reposting another user’s tweet to one’s own followers and marking as a favorite, indicating appreciation of a tweet, allow for further interaction between users and promotion of content.

Previous research has successfully used social media meta-data to describe the use and perceptions of health topics such as the use of little cigars and cigarillos [9], breast cancer [10], and pediatric obesity [11]. One of the characteristics of social media is its interactivity and the potential to engage a broad range of users in a dynamic conversation [8]. Previous health care–related social media research suggests that as recently as 2012, tweets made by state health departments lack the user engagement component, decreasing content impressions and potentially interest and dissemination power [12]. Number of retweets and followers on Twitter have been used previously as a proxy for interactivity [13].

The purpose of this study was to (1) identify the existing consumer and professional information about HF on social media and (2) identify RDNs’ and physicians’ attitude toward the use of social media to gather professional information and disseminate that to patients. Whereas other authors have suggested using mixed methods within Twitter content analysis, our study was primarily quantitative in the methods related to aim 1 and primarily qualitative in the methods related to aim 2 [14]. By using this approach, we were able to establish in aim 2 why we observed few health care professional voices discussing HF on Twitter in aim 1.

## Methods

### Design and Ethical Approvals

The methods were divided into two parts. Part 1 (Twitter content analysis) involved conducting content analysis of tweets related to HF. Part 2 (Health care provider interviews) involved conducting phone interviews with RDNs and physicians to identify health professionals’ attitude toward the use of social media for the communication of general health information and information specifically related to the EBNPG for HF. Both parts were reviewed and approved by the American Academy of Family Physicians Institutional Review Board. Part 1 was approved as an exempt project and follows the guidelines set forth by the European Society for Opinion and Marketing Research (ESOMAR) stating that public postings on social media sites may be used for research when identifiable information is protected and is consistent with the Twitter Terms of Service [15,16]. Part 2 was approved as human subjects research utilizing a verbal consent process. The 2 methods were designed in tandem but completed sequentially—the interview questions were written prior to the content analysis, but not conducted until after the Twitter analysis. Care was taken to keep the Part 2 interviewers blinded to the results of Part 1. The codes used in Part 1 were considered for Part 2 interview coding but mostly were not found to be relevant.

### Part 1: Twitter Content Analysis

Our method was loosely based on the one described by Step et al and similar to that described by Harris et al, particularly in the use of tracking a single hashtag [9,11]. Tweets that included “#heartfailure” were downloaded from Twittonomy. Twittonomy is a subscription Twitter aggregation service that allows the purchase of tweets and their associated metadata including username, hashtags, date posted, number of favorites and retweets. The download for this project was created on Tuesday, May 5, 2015 and included data from the previous 9 days, which included HF Awareness Day. “#Heartfailure” was selected for analysis based on surveillance of Twitter and use of analytics website (symplur.com) demonstrating that this was the most frequent hashtag applied to relevant messages. #HF, #CHF, #congestiveheartfailure, and #LVHF were also included in the surveillance but were not selected for download and analysis due to infrequent use.

Using a directed content analysis approach, one investigator (RKH) created a codebook with proposed codes and definitions (Table 1) before examining the tweets [14]. A second investigator (TMW) reviewed the codebook and suggested changes and additions that were made based on consensus among the 2 investigators. Once the codebook was edited, reviewed, and approved, both investigators individually coded the first 10% (29/294) of the downloaded tweets identified as original (excluding retweets). Their answers were compared and the final codes for each tweet were determined based on consensus. They also discussed and agreed upon whether new codes were needed, and changes to definitions of the existing codes. Tweets were viewed on the Twitter platform, which allowed viewing of any pictures that were included in the tweet (pictures were not included in the Twittonomy download), as well as profile information about the user who posted the tweet. Information about the user was used to determine the source and audience. If a tweet included a link to content, the initial posted link was opened and assessed as part of the content; however, coders did not open additional links from that page. A third investigator (JKA) who was trained to use the revised codebook, coded the first 10% (29/294) of tweets, and compared answers with the key created by RKH and TMW. The remaining 90% (265/294) of tweets were coded individually by an investigator (JKA). A 10% (29/294) random sample of these tweets was assessed by RKH and compared with the assessments of JKA. Discrepancies were noted and discussed. Of the 180 codes applied to the random sample of 29 tweets, 5 (2.8%) were removed after discussion and 12 (6.66%) were added, which was considered adequate agreement. The retweets were not coded. Codes were not mutually exclusive. The Twittonomy data indicated that how many times an original tweet had been retweeted or marked as a favorite as well as the number of followers a user had; these metrics were used to assess interest in a tweet.

If a user handle was listed at the beginning of a tweet, this was considered to be directed to that specific user. If a user handle was used at the end of the tweet, the named individual was considered to be a user who was related to the tweet. If a tweet varied only in the user handles listed, then it was coded identically to the original tweet. The recipients of these user-directed messages were considered in the audience assessment; the author’s profile was also used for assessment of the audience. Messages that were coded as irrelevant content did not have source or audience codes applied; therefore the N for these analyses is lower.

Once the codes were applied, the number of original tweets with each code was quantified. Since more than one code could be applied to a tweet, frequencies exceed 100. Because one individual’s posts (hereafter, frequent user) represented 84 of the tweets (28%), a post-hoc sensitivity analysis was performed to determine whether the proportion of tweets with each code varied when the frequent user’s tweets were excluded from the entire sample. The next most frequent user only posted 14 messages, making the frequent user a clear outlier. The sensitivity analysis was performed using one sample *t*-tests comparing the frequency of each code with and without the frequent user’s tweets.

Using the entire sample of tweets (N=294), differences in mean retweets and favorites for each message were compared using independent sample *t*-tests, based on whether each content code was applied. In addition to the number of times a specific tweet was marked as a favorite or retweeted, the mean number of followers for the user who had posted the message was compared for each tweet based on the source and audience codes using independent sample *t*-tests. In both cases, Levene’s test for equality of variance was used and if it was statistically significant, a *t*-test that did not assume equality of variance was used. The number of retweets, favorites, and followers were considered as a measure of interactivity [13].

Analysis was performed in SPSS version 20.0 (IBM Inc) and significance was set as *P*<.05.

### Part 2: Health Care Provider Interviews

Interview participants were recruited via emails to the Academy’s Dietetics Practice Based Research Network (n=1815) and the American Academy of Family Physicians’ (AAFP) Research Committee (n=10), AAFP Foundation grant reviewers (n=30), Commission on Health of the Public and Sciences (n=22). Participants were required to be physicians or RDNs and see outpatients with HF. Forty-two RDNs replied to the email and indicated they were interested in participating, and the first 10 who were eligible were selected. A total of 7 physicians, 3 physician assistants or nurse practitioners, and 2 family medicine researchers (PhDs) replied to the email and indicated they were interested in participating—only the 7 physicians were eligible and were scheduled.

Sixteen individuals (6 physicians, 10 RDNs) participated in an interview. One eligible physician did not attend the scheduled interview and opted not to reschedule. After obtaining verbal consent to participate in the study, participants were individually interviewed by trained interviewers via telephone using a semistructured interview protocol developed to assess knowledge, use, importance, and accuracy or validity of the Academy’s HF EBNPG. The interview also explored provider and health care system, use of technology, and social media with HF patients to communicate general health or nutrition information. Questions about personal use of technology and social media were included in an effort to understand health care providers’ familiarity and comfort level with these communication methods and to determine whether professional attitudes were shaped by personal use patterns. Interview participants were compensated US $150 for participating in the study. Interviews were recorded and transcribed verbatim, although filler words (eg, umm) were removed in the exemplar quotes presented here. In an effort to increase accuracy of transcriptions and thus reduce error, each interviewer transcribed only the interviews that he or she conducted. Transcripts were analyzed ethnographically using MaxQDA version 11 (VERBI GmbH) to identify themes in participants’ responses to each set of questions presented in the interview. The unit of analysis was each participant’s response to a specific question. Question responses could have more than one theme applied.

## Results

### Part 1: Twitter Content Analysis

The Twittonomy download included 298 original and 324 retweets that included #heartfailure. Between downloading and coding, 4 tweets had been deleted. Thirty-seven tweets were identical to previously coded tweets; these were included in the sample and received the same codes as the original tweets. A total of 728 content, 365 source, and 287 audience codes were applied, representing 2.47 (SD 1.61), 1.24 (SD 0.66), and 1 (SD 0.59) codes in each category per tweet, respectively.

The most frequent content code was “HF awareness” (166/294, 56.5%), followed by “patient support” (97/294, 33.0%; Table 1). However, the frequency of these content codes was strongly influenced by the content of the frequent user. The frequency decreased to 45.5% (95/209) and 10% (21/209), respectively when the frequent user’s tweets were removed (*P*=.001). The second most frequent code without the frequent user’s tweets was “HF research” (81/209, 38.8%). The most frequent source code among all tweets when the the frequent user was included in the sample was “professional, government, patient advocacy organization, or charity” (112/277, 40.4%), followed by “patient/family” (105/277, 37.9%). Without the frequent user’s tweets the most frequent source code was “other” followed by “professional, government, patient advocacy organization, or charity” (81/192, 42.2% and 70/192, 36.5%, respectively) (Table 1). Users coded with the “other” source code included medical journals, health news services, and health websites like WebMD. The frequency of the source codes “patient and family” and “other” were statistically different based on the inclusion or exclusion of the frequent user’s tweets. The most frequent target audience codes were “unable to identify” (111/277, 40.1%) and “other” (55/277, 19.9%). “HF nutrition” was rarely a theme of the messages (<10%) and RDNs were infrequent tweeters (1 message). “Other” target audiences included political figures and health advocates.

**Table 1:** Codes applied to tweets with the hashtag “#heartfailure” in a landscape analysis of social media content related to nutrition and heart failure.

Category	Code	Definition	Number of tweets (%) with code n (%)	Number of tweets (%) with code except frequent user’s tweets n (%)	*P* value of one sample *t*-test by comparing frequency with and without frequent user’s tweets
Message content –What is the tweet discussing?			N=294	N=209	
	Awareness	Raising awareness of heart failure including its prevalence and/or risk factors	166 (56.5%)	95 (45.5%)	.002
	Patient support	Messages of support for patients with heart failure or support systems	97 (33.0%)	21 (10.0%)	<.001
	HF^b^ research	Research related to HF	81 (27.6%)	81 (38.8%)	.001
	HF symptoms	Symptoms of HF such as fluid overload and shortness of breath. Also includes side effects and related conditions	79 (26.9%)	51 (24.4%)	.40
	HF outcomes	Outcomes of HF or HF treatments or research, may include mention of hospital re-admissions	68 (23.1%)	68 (32.5%)	.004
	HF management	Standard strategies for management of heart failure not specific to nutrition or exercise or medication. Novel strategies will more often fall under research.	58 (19.7%)	58 (27.8%)	.01
	Event	Advertising a specific event either for fundraising or a course opportunity	47 (16.0%)	28 (13.4%)	.27
	HF medication	Medications used to treat HF	34 (11.6%)	34 (16.3%)	.07
	Exercise	Exercise for HF including cardiac rehab programs	30 (10.2%)	14 (6.7%)	.045
	Fundraising	Raising money for heart failure research or charity	23 (7.8%)	6 (2.9%)	<.001
	Not relevant	Message is not relevant to HF	17 (5.8%)	17 (8.1%)	.22
	Other	Other message content related to HF	15 (5.1%)	15 (7.2%)	.25
	HF nutrition (general or other)	Nutrition requirements or restrictions for HF or mention of a dietitian in relation to HF. Use only if the subsequent specific codes cannot be used.	12 (4.1%)	12 (5.7%)	.31
	HF sodium restriction^a^	Sodium restrictions for patients with HF	10 (3.4%)	10 (4.8%)	.35
	HF fluid restriction^a^	Fluid restriction for patients with HF	6 (2.0%)	6 (2.9%)	.71
	HF energy needs^a^	Energy need for patients with HF	1 (0.3%)	1 (0.5%)	.71
	HF dietary supplements^a^	Dietary supplement products for patients with HF	1 (0.3%)	1 (0.5%)	.71
	HF alcohol^a^	Use or misuse of alcohol in the context of HF	0	0	
	HF protein needs^a^	Protein needs for patients with HF	0	0	
Source–who posted the tweet? Identified using information in the tweet or the author’s profile			n=277	n=192	
	Professional, government, patient advocacy organization, or charity	Non-profit, charity, government organization, dedicated to a disease or condition or professionals related to that condition. Generally but not always specific to HF.	112 (40.4%)	70 (36.5%)	.26
	Patient or family	Patient with HF or family member of a patient with HF, or someone who identifies as being at risk of HF	105 (37.9%)	28 (14.6%)	<.001
	Other	A poster who has identifiable characteristics that are not described above	81 (29.2%)	81 (42.2%)	<.001
	Provider or hospital group	A hospital or medical care organization that includes more than one practitioner	25 (9.0%)	25 (13.0%)	.10
	Individual physician	A physician who is posting on his own rather than as part of an organization	16 (5.8%)	16 (8.3%)	.21
	Industry	Entity selling a product relevant to HF	14 (5.1%)	14 (7.3%)	.25
	Other individual provider	Another health care professional who is posting on his own rather than as part of an organization	8 (2.9%)	8 (4.2%)	.38
	Unable to identify	The characteristics of the poster cannot be determined	3 (1.1%)	3 (1.6%)	.53
	Individual RDN	A dietitian who is posting on their own rather than as part of an organization	1 (0.4%)	1 (0.5%)	.82
Target audience–who is the message’s intended reader? Identified using information in the tweet or hashtags			n=277	n=192	
	Unable to identify	The characteristics of the audience cannot be determined	111 (40.1%)	81 (42.2%)	.64
	Other	Audience who has identifiable characteristics that are not described above	55 (19.9%)	25(13.0%)	.004
	Patient or family	Patient with HF or family member of a patient with HF, or someone who identifies as being at risk of HF	49 (17.7%)	44 (22.9%)	.10
	Other individual provider	Another health care professional who is posting on his own rather than as part of an organization	23 (8.3%)	10 (5.2%)	.05
	Professional, government, patient advocacy organization, or charity	Nonprofit or government organization dedicated to a disease or condition or professionals related to that condition. Generally but not always specific to HF.	18 (6.5%)	14 (7.3%)	.70
	Individual physician	A physician who is posting on his own rather than as part of an organization	21 (7.6%)	15 (7.8%)	.95
	Provider or hospital group	A hospital or medical care organization that includes more than one practitioner	7 (2.5 %)	7 (3.6%)	.41
	Industry	Entity selling a product relevant to heart failure	2 (0.7%)	2 (1.0%)	.65
	Individual RDN	A dietitian who is posting on his own rather than as part of an organization	1 (0.4%)	1 (0.5%)	.82

^a^Indicates the recommendations from the Academy’s EBNPG for HF on that topic

^b^HF: heart failure.

Codes applied to tweets with the hashtag “#heartfailure” in a landscape analysis of social media content related to nutrition and heart failure are presented in Table 1. Codes were applied for tweet content, source, and audience. Themes are presented in descending order of frequency. To test the effect of one very active patient tweeter on the frequency of codes (frequent user), one-sample *t*-tests were used to compare the frequency of each code in the sample of tweets that did and did not include his posts.

Number of followers varied widely and no statistically significant patterns were discerned based on source or target audience (data not shown). Significant differences were found in the popularity of tweets with or without the content code of “HF research.”(Table 2) Tweets with the code had more favorites (1 [SD 1.3] vs 0.7 [SD 1.3], *P*=.049), but fewer retweets (0.7 [SD 1.2] vs 1.3 [SD 2.0], *P*=.003). Tweets with the content code of “HF outcomes” were also less likely to be retweeted than tweets without this content code (0.6 [SD 1.6] vs 1.2 [SD 1.9], *P*=.023). Tweets with the content code “not relevant” were also less likely to be marked as a favorite. Tweets with the source code “professional, government, patient advocacy organizations, or charities” were significantly more likely to be marked as a favorite and retweeted than those without this source code (1.2 [SD 1.4] vs 0.8 [SD 1.2], *P*=.026), (1.5 [SD 1.8] vs 0.9 [SD 2.0], *P*=.026). No statistically significant differences were found for the target audience codes.

### Part 2: Health Care Provider Interviews

Demographic characteristics of interview participants are shown in Table 3. Physician participants were somewhat older (mean 47.8 years) and reported more experience (mean 19.6 years) than RDNs (mean 40.2 years and 13.5 years, respectively). The overall sample were predominately white and female. The physician sample was somewhat more diverse than the RDNs.

Awareness of the Academy’s HF EBNPG was low among both physician and RDN interview participants. RDNs were more likely to report being somewhat or fairly familiar with the Academy’s HF EBNPG; one was very familiar. RDNs were also more likely to report being familiar with and using guidelines from the American Heart Association (AHA). Participants from both professions indicated that they believe guidelines are useful in caring for HF patients. They focused on the sodium and fluid restriction components of nutrition guidelines.

If it (Academy HF EBNPG) has anything to do with salt, but I don’t know about any other nutritional guidelines so if it has to do with salt intake and so forth then yes, I would assume it would be helpful.

Physician

Two RDN participants were able to identify that the Academy’s HF EBNPG recommends a stricter sodium restriction (2000 mg/day) [1] when compared to the AHA guideline, which simply states that for stage C class 3A HF, “sodium restriction is reasonable for patients with symptomatic HF to reduce congestive symptoms,” without specifying a target intake [17].

Interview participants from both the professions reported the difficultly of behavior change for their patients. Physicians were likely to report not having time to review detailed diet information during office visits or their lack of nutrition-specific training. Although this might be expected to be associated with RDN referrals, physicians also identified many barriers to RDN referrals for patients including inability to pay and the high cost due to lack of insurance coverage for nutrition counseling. While both professions agreed on the need for an inter-professional team approach to HF, many reported that patient care was in fact disjointed:

I’m in one area, cardiology is in another separate building...some of our other HF areas are in whole other areas. So that’s where some of that disjointedness comes together, and so having that continuity, and then if you look at across system you have different recommendations coming about...then I finally see the patient and I’m re-educating the patient, that is then totally confused on what they should or should not do.

RDN

RDNs were more likely to report using Facebook as a personal past-time as compared with physicians. RDNs were also more likely to report personal use of other social media channels such as Instagram and Pinterest. Personal use of Twitter was reported more commonly by physicians. Most interview participants reported professional use of technology such as email, the Internet, Web conferencing, listservs, electronic medical records (EMR) and patient portals. Participants described their use of social media and technologies to network with other professionals and stay up to date with new information:

Twitter is a really good useful tool in keeping up with medical literature, because if you follow the right people, both sort of journalists, and medical professionals, you can often get very good information or links to very good information. So that’s, that’s something I use quite frequently, probably daily. At least I’m checking Twitter to see what new developments there are.

Physician

When deciding what information to read online, participants cited credibility of the source and interest in the topic as the most important factors. One RDN identified that the host suffix was important in determining credibility, for example the “.edu” domain may be more credible than “.com”. Another RDN reported that she often accesses information if the title “sparks” her interest:

(I am more likely to click on...)Something that just might be a little bit different or be controversial or something different from mainstream that might be said that might get, you know, I’d, I’d be more interested in that.

RDN

Participants were adamant about not using or not being permitted to use social media or certain other technologies to communicate with patients, citing legal, and ethical considerations related to the Health Insurance Portability and Accountability Act (HIPAA) and privacy:

From the patient’s side...I feel like they give their implied consent if they’re going to post their personal health information on social media. From the medical professional side, I feel like the appropriate thing is sort of, not to, sort of, if somebody wants to engage in a conversation about their personal health issues, you should guide them to another channel, instead of over tweets and replies or Facebook posts; things that are open. Because then, that does create potential ethical issues about sharing that private information.

Physician

Most patient communication (eg, lab results), was handled through patient portals. Many participants reported that their institution communicated general medical information to patients through social media channels, including Facebook and Twitter, and other digital communications channels such as texting:

So, both the institution, the university has, you know, an account that posts health information now and then. And then our department also has a Facebook account and a Twitter account that occasionally will post links to articles or things with health information.

Physician

Some participants described how they used technologies during office visits to steer patients toward credible information. One RDN commented that she shared recipes, products, and “tips and tricks” with patients via Pinterest. A physician described using social media to communicate general health information to patients, but that this was not targeted to his patients specifically. Interview participants also noted that Facebook, Twitter, and other digital communications were often used to promote or advertise educational opportunities for patients. Interview participants were concerned about the credibility of information posted on social media, including in some cases the credibility of information posted by their own institutions when the social media managers were not medical professionals:

My only concerns are sometimes the person who is in charge of posting to those (social media) accounts does not have actually a medical background, at least at our department level. I’m not sure who does it at the university level, but there are occasionally things, posted or shared that I feel like are maybe not the most evidence based, or the most accurate, and so I do have concerns from that stand point.

Physician

Interview participants believed that social media could be effective channels to communicate health information to HF patients but were concerned about patient access to the Internet and use of technology. RDN interview participants reported that their HF patients tended to be older adults. Consequently, RDN interview participants believed patients were unlikely to be utilizing technology or social media:

Most heart failure patients are in their late 60’s, 70’s and above...A lot of those folks don’t, you know, it’s not their generation (to use social media).

RDN

Two RDNs discussed how social media might be used to provide support groups and disseminate health information to their patients with heart failure. One RDN suggested an “online community” that would be moderated by a health professional or other expert might be useful approach to engage patients with heart failure. Likewise, another RDN suggested that a private Facebook group might be utilized to support and educate HF patients.

To ensure patient privacy, interview participants believed that social media can only be used to provide general health information. However, participants pointed out that patients are more likely to respond to information that is individualized to them:

They’re less likely to respond to things that aren’t specifically directed to them, you know, like I said, so, so putting things out on Twitter is probably less effective than say emailing to them, or you know having, having like an App that they can download. I feel like that might be...a good way is if there was an App that the patients could access you know directly on their own device that sort of integrated recommendations and things like that with how they’re tracking their own health information. That might be helpful, but I feel like things that aren’t personalized are less helpful.

Physician

Similarly, interview participants recognized that general information on social media might help to raise awareness of guidelines among patients but that this would not necessarily translate into action or behavior change, which probably requires more personalized information:

So, if the idea is to get patients to recognize the guidelines exist, and perhaps get them to look at it, then whether it be Twitter or Facebook or something like that, I think you can make people aware. However, to actually get people to adopt those lifestyle choices, then I think it works better to come electronically, from their provider through the electronic portals most of us have with their electronic health records.

Physician

**Table 2:** Comparison of mean favorites and retweets for messages with each content, source, and audience code versus messages without these codes in a sample of tweets using the hashtag “heartfailure” compared with independent samples *t*-tests.

Category	Code	Favorites				Retweets		
Message content		Mean (SD) with code	Mean (SD) without code	*P* value for independent samples using *t*-test	Mean (SD) with code	Mean (SD) without code	*P* value for independent samples using *t*-test
	HF^b^ symptoms	0.8 (1.4)	1.0 (1.3)	.234	0.8 (1.7)	1.2 (2.0)	.13
	HF management	0.1 (1.6)	0.9 (1.2)	.92	0.9 (1.7)	1.1 (1.9)	.48
	Exercise	0.8 (1.0)	0.9 (1.3)	.66	1.1 (1.3)	1.1 (2.0)	.44
	Awareness	1.0 (1.2)	0.8 (1.4)	.30	1.1 (1.6)	1.0 (2.2)	.61
	Fundraising	1.4 (1.0)	0.9 (1.3)	.11	1.3 (1.2)	1.1 (1.9)	.57
	Event	1.2 (1.2)	0.9 (1.3)	.08	1.2 (1.4)	1.1 (2.0)	.69
	Patientsupport	1.0 (1.1)	0.9 (1.4)	.43^a^	1.1 (1.5)	1.1 (2.1)	.82
	HF medication	0.7 (1.3)	1 (1.3)	.22	0.7 (1.5)	1.2 (1.9)	.15
	HF research	1 (1.3)	0.7 (1.3)	.049	0.7 (1.2)	1.3 (2.0)	.003^a^
	HF outcomes	0.71 (1.2)	0.7 (1.4)	.10	0.6 (1.6)	1.2 (1.9)	.023
	HF nutrition (general or other)	0.5 (1.2)	0.1 (1.3)	.24	0.4 (1.1)	1.1 (1.9)	.21
	HF energy needs	0	0.9 (1.3)	.47	0	1.1 (1.9)	.57
	HF fluid restriction	1.0 (2.0)	0.9 (1.3)	.90	1.2 (2.9)	1.2 (1.9)	.92
	HF sodium restrictions	1.0 (2.2)	0.9 (1.3)	.87	1.0 (2.2)	1.1 (1.9)	.88
	HF dietary supplements	6.0	0.9 (1.3)	<.001	2	1.1 (1.9)	.63
	Other	1.1 (1.8)	0.92 (1.3)	.68	1.2 (2.1)	1.1 (1.9)	.93
	Not relevant	0.4 (0.6)	1.0 (1.3)	<.001^a^	0.4 (0.8)	1.1 (1.9)	.10
Source	Patient or family	1.1 (1.2)	0.9 (1.4)	.33	1.1 (1.2)	1.2 (2.3)	.61^a^
	Industry	1.1 (1.9)	0.96 (1.3)	.73^a^	1.1 (2.4)	1.1 (1.9)	.90
	Professional, government, patient advocacy organization, or charity	1.2 (1.4)	0.8 (1.2)	.026^a^	1.5 (1.8)	0.9 (2.0)	.026
	Provider or hospital group	0.9 (1.4)	1.0 (1.3)	.85	0.8 (1.5)	1.2 (2.0)	.43
	Individual physician	1.2 (1.5)	1.0 (1.3)	.49	2.0 (4.7)	1.1 (1.6)	.44^a^
	Individual RDN	0	1.0 (1.3)	.46	0	1.1 (1.9)	.56
	Other individual provider	0.6 (0.7)	1.0 (1.3)	.46	0.8 (1.2)	1.1 (2.0)	.57
	Other	0.7 (1.4)	1.1 (1.3)	.05	0.9 (2.4)	1.3 (1.7)	.12
	Unable to identify	0.7 (1.2)	1.0 (1.3)	.69	0	1.2 (1.9)	.31
Target audience	Patient or family	1.2 (1.9)	0.93 (1.2)	.40^a^	1.53 (2.1)	1.1 (1.9)	.15^a^
	Industry	2.0 (1.4)	1.0 (1.3)	.27	2.0 (1.4)	1.1 (1.9)	.52
	Professional, government, patient advocacy organization, or charity	1.3 (1.0)	0.9 (1.3)	.22	1.4 (2.4)	1.1 (1.9)	.56
	Provider or hospital group	1.7 (1.8)	1.0 (1.3)	.31	2.0 (2.6)	1.1 (1.9)	.13
	Individual physician	1.2 (1.3)	1.0 (1.3)	.33	0.9 (1.6)	1.2 (2.0)	.57
	Individual RDN	5	1.0 (1.3)	.002	7	1.1 (1.9)	.002
	Other individual provider	0.5 (0.7)	1.0 (1.3)	.09	0.5 (1.0)	1.2 (2.0)	.09
	Other	0.8 (1.1)	1.0 (1.4)	.35	0.8 (1.1)	1.2 (2.1)	.05^a^
	Unable to identify	0.9 (1.1)	1.02 (1.0)	.61	1.1 (1.6)	1.2 (2.1)	.43

^a^Indicates that Levene’s test for equality of means was statistically significant at *P*<.05 and so the *t*-test did not assume equality of variance.

^b^HF: heart failure.

**Table 3:** Demographic characteristics of interview participants. Age and experience are reported as mean and standard deviation; other characteristics are n and %.

Demographic characteristics		Physician (N=6)	Registered dietitian nutritionist (RDN) (N=10)	Combined (N=16)
**Gender, n (%)**				
	Male	4 (67%)	0 (0.0%)	4 (25%)
	Female	2 (33%)	10 (100%)	12 (75%)
**Ethnicity or race, n (%)**				
	White	5 (83%)	9 (90%)	14 (88%)
	Black	0 (0.0%)	0 (0.0%)	0 (0.0%)
	Asian	1 (17%)	1 (10%)	2 (13%)
	Hispanic or Latino	0 (0.0%)	0 (0.0%)	0 (0.0%)
	Other	0 (0.0%)	0 (0.0%)	0 (0.0%)
	Age^a^ , mean (SD)	47.8 (10.8)	40.2 (8.6)	42.4 (9.9)
	Years of experience^b^ , mean (SD)	19.6 (8.6)	13.5 (10.5)	15.5 (10.3)


^a^Two physicians declined to report age.

^b^One physician declined to report years of experience.

## Discussion

### Principal Findings

Together, these findings support a role for health providers and organizations to use interactive social media —such as Twitter — to complement patient voices and provide important information to increase knowledge (dissemination) on focused health topics. This study shows that individual users are critical in shaping the content of social media, so if health care providers and their professional organizations are not active on social media, patient voices or nonexpert sources may dominate. Both individuals and organizations should attempt to provide evidence-based information to patients and colleagues, but with clear differentiation of the target audience. When a frequent user’s tweets were included in analysis, the “patient support” and “fundraising” content codes and the “patient” source code were most frequent. Without this frequent user’s tweets, “HF awareness” and “HF research” became the most frequent content codes. These data support Moorhead’s uses of social media for providing health information on a range of conditions, for health intervention, promotion, and education [6]. Using social media to facilitate dialogue between patients to health professionals was a limited use in both our quantitative and qualitative datasets, but patient to patient dialogue was seen more frequently.

Different types of organizations use social media channels to varying degrees and it is likely that the preference for different channels varies over time as social media trends change [8]. For example, many hospitals use Facebook to provide health education information, recognize staff, and share hospital news, such as awards [18]. In our Twitter sample, journals and professional organizations were well represented and had success in interactivity as measured by favorites and retweets, indicating that social media users are interested in HF content from these resources. It is unclear whether journals are targeting the public or medical professionals. Our findings are similar to those in childhood obesity, which suggest that there is room for increased credible, evidence-based information from health organizations on Twitter [11].

Recent research examining guidelines from the American Academy of Neurology (AAN) indicated that social media dissemination did not supplement knowledge gains seen with traditional dissemination methods among physicians or patients [19]. This research used paid advertisements on social media sites, so it is unclear whether the results would be different if social media messages were used directly instead of using advertising placed on the sites [19]. The AAN study authors propose that their results are explained by a ceiling effect, in which individuals who already feel they know the information, perhaps from traditional dissemination methods, will not make any effort to learn more [19]. Sense of competence (the belief that one is already an expert in the content) frequently impedes dissemination of new information and may contribute to this ceiling effect [20]. Similarly, interview participants in our study indicated that they were much more likely to investigate or read more about content that was new, controversial, or different. Framing information in these ways may represent an opportunity to break through the “noise” of information overload. It is possible that the existing information on social media from professional organizations is adequate and that more information would meet this ceiling. However, much of the professional organization content was directed at patients or families; so directing this content to health care providers may be a new opportunity. Our interviews showed the participants willingness to use social media to obtain information for themselves.

### Limitations

One theme that was unique to interviews and did not appear in the content analysis was participants’ belief that age or socioeconomic status limited access to and use of social media. Given that the Pew Internet Report demonstrated widespread use of the Internet and social media across age and class groups, health professionals may need to be reeducated about who could benefit from social media [2]. Although social media use was lower among those aged 65 years and older, more than one in 3 of these individuals were using social media [2]. Assessing Internet availability and literacy as a component of health literacy may become necessary in order to customize educational materials and other resources for patients’ needs. In addition, it was clear from our interviews that health professionals have significant concerns about privacy and social media, many of which have trickled down from their employers. Whereas there are privacy concerns, previous research has demonstrated that these may be overblown [18]. These incorrect assumptions about who uses social media and how it can be used while protecting patient privacy could prevent valuable information from reaching patients who need it. This is a challenge that was pointed out by Gholami-Kordkheili et al in their review of social media and medical professionalism—while social media has the potential to improve access to care by decreasing geographical barriers, access to the technology is required in order to reap these benefits [7]. Professional organizations may have a role in providing continuing education on the topic of social media use in order to overcome this misperception and provide guidance on using social media while protecting privacy.

We had previously demonstrated that RDNs are willing to take surveys about Academy EBNPG even when they are not familiar with the content; this is a method of learning new information, suggesting that surveys and quizzes may be novel dissemination strategies [21]. These strategies offer an element of interactivity and could be linked to social media posts. Thus far, attempts to use social media as a health information dissemination strategy have not used interactive components but rather use social media as an extension of traditional information sharing strategies. This failure to capitalize on the true Web 2.0 nature of social media could be the reason for poor uptake from social media dissemination strategies tested in the past and should be a focus of future work.

The Twitter analysis is limited by the use of a single hashtag to identify relevant tweets. Kim et al have suggested that keyword searches be used instead of hashtags, which may lead to a more sensitive and specific search [22]. Our use of surveillance to identify the hashtag on which we focused overcomes this to some extent, but we did not assess the sensitivity and specificity of our search results. Retweets, favorites, and number of followers were stand-ins for the interactivity of Twitter on the level of individual message themes, posters and audiences; however, it may be more appropriate to measure the interactivity of a specific user [13].

In many cases, it was challenging to identify the target audience of a specific tweet or a user in the content analysis. We used only profile information of the user posting the message to define the (intended) audience; however, methods have been developed to use the recent tweets from followers to define the interests of the actual audience [23]. We were also unable to use the data-mining techniques developed by Xu et al to describe the race and ethnicity of users and further describe both the posters and their audience [24]. Organizations or individuals using Twitter to disseminate evidence-based information should be mindful of cultivating a clear intended audience, as messages will likely differ when directed toward professionals versus patients. Some organizations have different accounts to disseminate their professional-oriented content versus patient-oriented content, which is one strategy to clarify the intended audience. In creating a social media strategy and evaluation plan, organizations should clarify whether their goal is dissemination (knowledge) or implementation (execution) of information. As expressed in the interviews, dissemination may be a reasonable goal for social media campaigns, while implementation may not be.

Despite the findings that most state health departments were using social media for one-way information sharing, the audience for these messages were unclear, similar to our findings in the content analysis [12]. Posting done by hospitals on Facebook were also found to share information more than interaction, with only 27% responding to comments posted on their page [18]. Together, these results lend validity to the concept, reflected in interviews, that social media was used as a dissemination rather than an implementation or engagement tool. However, Cameron et al were able to use Facebook to change actions, with their novel intervention to increase the registry of organ donors [25]. This was a one-time action rather than a behavior or lifestyle change, but it demonstrates that the interactivity of social media can be used to go beyond dissemination to implementation [25].

The frequent user clearly modified frequency of themes in the sample tweets; therefore, the content analysis may not be representative. It is unclear whether the inclusion of HF awareness day in our sample influenced the results. The timeframe for our gathering of Tweets was short because this was an initial exploration of the research question. The small sample size of Tweets limits generalizability and future research should use a longer collection period to ensure a larger number of messages are collected. Previous research indicates that there is a spike in the related Internet searches during breast cancer awareness month, but a smaller or no spike during lung or prostate cancer awareness events [24]. Given the media publicity for breast cancer awareness month, for which there is no HF equivalent, we would anticipate that HF awareness would be more similar to lung or prostate cancer, and therefore the influence on Twitter activity is likely to be low. Fundraising was a frequent topic in breast cancer awareness month tweets, similar to our sample [10]. It is interesting that the breast cancer analysis did not focus on the outcomes of research, but rather on fundraising for research [10].

The qualitative portion of this study built on the content analysis to understand how health professional attitude toward social media might explain the gaps in information identified in the content analysis, but is limited by the small sample of interview participants. The sample of RDNs and physicians who were interviewed may not be representative of all practitioners in their profession, in particular because we focused on a specific group—those who provide primary care to outpatients with HF. In addition, nurses and physician extenders may have an expanding role in taking care of patients with HF, and we did not interview any of these professionals because at the time of our initial grant application, this shift had not yet occurred. Because of the limited demographics collected from the interview participants, we are unable to compare them with their professions as a whole. The RDNs may be more representative than the physicians because of the use of a large Practice Based Research Network for recruiting [26,27]. In addition, more RDNs were interested in participating than were needed, leading to semirandom selection of the participants. We did not attempt to validate the interview responses through methods such as member checking. We were unable to determine whether the use of social media or knowledge of the EBNPG varied based on demographic or practice characteristics of the interview participants.

### Conclusions

This study adds to a growing body of literature on the use of social media to disseminate health information. There are clear gaps in the current HF content on social media, which health organizations can fill, using information in evidence-based practice guidelines and targeting both patients and providers, although under separate cover. Health care providers have adopted social media as a way to gather information but are more skeptical of its use in their own communications with patients, which may be the reason that content from health care providers and their organizations are limited. Because the literature on the effectiveness of social media as a tool for health information dissemination is mixed, any social media campaigns should have a rigorous evaluation plan to continually assess this evolving digital communications strategy. The effectiveness of social media for dissemination of information to the public must also be demonstrated to show whether there is value to the provider in assuming the legal or liability risks of these communication strategies.

## Abbreviations

AHA: American Heart Association
AAFP: American Academy of Family Physicians
CHF: congestive heart failure
EBNPG: Evidence-Based Nutrition Practice Guideline
EMR: electronic medical record
HF: heart failure
LVHF: Health Insurance Portability and Accountability Act
HIPAA: left ventricular heart failure
RDN: registered dietitian nutritionist
